# Mechanical Strength and Chloride Ions’ Penetration of Alkali-Activated Concretes (AAC) with Blended Precursor

**DOI:** 10.3390/ma15134475

**Published:** 2022-06-24

**Authors:** Patrycja Duży, Marta Choinska, Izabela Hager, Ouali Amiri, Jérôme Claverie

**Affiliations:** 1 Chair of Building Materials Engineering, Faculty of Civil Engineering, Cracow University of Technology, 31-155 Cracow, Poland; izabela.hager@pk.edu.pl; 2 Research Institute in Civil and Mechanical Engineering GeM—UMR CNRS 6183, IUT Saint-Nazaire, Nantes University, 44600 Saint-Nazaire, France; marta.choinska@univ-nantes.fr (M.C.); ouali.amiri@univ-nantes.fr (O.A.); jerome.claverie@univ-nantes.fr (J.C.)

**Keywords:** chloride ions’ penetration, chloride ions’ diffusion, alkali-activated concrete (AAC), mechanical strengths

## Abstract

The purpose of this study was to investigate the properties of hardened alkali-activated concrete, which is considered an eco-friendly alternative to Portland cement concrete. In this paper, the precursors for alkali-activated concrete preparations are blends of fly ash and ground-granulated blast-furnace slag in three slag proportions: 5%, 20%, and 35%, expressed as a percentage of fly ash mass. Thus, three concretes were designed and cast, denominated as AAC5, AAC20, and AAC35. Their physical and mechanical characteristics were investigated at 28 and 180 days, as well as their properties of chloride ion transport. The modified NT BUILD 492 migration test was applied to determine the chloride ions’ penetration of the alkali-activated concretes. Improvement of mechanical strength and resistance to chloride aggression was observed with ground-granulated blast-furnace slag content increase in the compositions of the tested concretes. Mercury intrusion porosimetry tests provided insight into the open pore structures of concretes. A significant decrease in the total pore volume of the concrete and a change in the nature of the pore diameter distribution due to the addition of ground granulated blast furnace slag were demonstrated.

## 1. Introduction

Cement production alone contributes between 5% to 7% of anthropogenic CO_2_ emissions worldwide [1]. Alkali-activated concrete (AAC) is an environment-friendly material and is presently accepted as an alternative to conventional concrete [2]. AAC utilizes industrial by-products to reduce CO_2_ emissions associated with cement production. Despite having been investigated for decades, the application of alkali-activated materials in construction is still very limited [3]. One major reason behind this slow and often underappreciated acceptance of AAC in industries is that there are limited reports related to AAC itself [4]. The work on material that can replace the Portland cement in recent decades has become the target of numerous studies. Due to successive regulations limiting carbon dioxide emissions [5], the intensification of research in this area has resulted in a growing base of theoretical [6] and practical [7,8,9,10] knowledge about alkali-activated binders. As presented by Mendes et al. [11], most of the research data refer to the properties, performances, and durability of alkali-activated pastes and mortars [12,13,14,15,16,17,18]. Alkali-activated concretes are well-accepted in the research community owing to their comparable or even better performances as cement substitutes [19]. However, there is a need for complementary studies with a view to future standardization for the aforementioned concretes.

AAC’s binders are based on aluminosilicate precursors activated by alkaline solution [6]. The most common sources of aluminosilicate mentioned in the literature are fly ash (FA), slag, kaolinite, mining wastes, etc., [20]. In the case of the activator, hydroxides and silicates of alkali metals (mainly sodium or potassium) are used [21,22]. Numerous factors determine the properties of the hardened binder and AAC including liquid/solid ratio, precursor composition [15], activator nature [23], fineness, and degree of crystallinity of precursor [24]. Curing conditions also have a major influence on the final material. Alkali-activated fly ashes need heat curing [25], which is a disadvantage for an eco-friendly approach. In the case of alkali-activated slag, curing at ambient temperature can be applied. However, the short setting time and poor workability of these mixes limit the proper process of casting [26,27]. The use of blended precursor may resolve the problem of curing, which is due to slower reactivity at ambient conditions of alkali-activated blended binders with a lower slag content [28,29]. This optimizes the setting time and the workability of the mixture without additional temperature curing [30]. This approach is increasingly recognized among researchers [3,9] and will be discussed in this paper.

The growing interest in this material has also brought many challenges for researchers. The implementation of the use of AAC requires the determination of a number of its properties, such as for cement concrete. For both Portland cement concrete and AAC, their most important characteristic is compressive strength. However, the influence of the environment on the durability of constructions forces us to define the resistivity of applied materials to aggressive environmental factors. Chloride ions e.g., from seawater or de-icing salts, are considered one of the most dangerous environmental factors for reinforced concrete constructions [31,32] due to corrosion of steel bars. Owing to the lack of testing standards dedicated to AAC, current tests of chloride ion penetration are performed according to the standards for Portland cement concretes. However, their results provide conflicting results. Using the methodology described by ASTM C1556 [33], Kupwade-Patil and Allouche [34] reported that fly-ash-based AAC has better resistance to chloride ion aggression than cement concrete. However, with the use of the same method, Babae and Castel [35] observed a similar performance for AAC based on FA to Portland cement concrete with the evaluation of their electrochemical performances. On the other hand, Olivia and Nikraz [36], in their research based on NT Build 443 [37], presented that FA-based AAC displayed higher values of chloride diffusion coefficient compared to concrete with a cementitious binder. Fan et al. [38] noted that slag addition in alkali-activated binder reduces chloride penetration into AAC. This has also been observed by Chen et al. [39]. The examples of the studies indicate the need for further study of the topic. In case of rapid chloride penetration tests, the result’s discrepancy can be multiplied because of additional conditions during the test e.g., electric voltage being applied [40,41]. Ideas for adapting cement concrete standards for AAC are also emerging. Noushini and Castel [42] proposed a reduction in electric voltage, supporting the idea with research on the electrochemical performance of AAC. The necessity of modifications was also observed by Aiken et al. [43].

The research presented in this paper covers the analysis of the influence of the composition of the precursor used on the strength properties of AAC and the penetration of chloride ions into the material. The tested blends of fly ash and ground granulated blast-furnace slag in three slag proportions, 5%, 20%, and 35%, expressed as a percentage of fly ash mass, and were denominated as AAC5, AAC20, and AAC35, respectively. Due to slag addition, the provided AACs could be cured in ambient conditions, without any heating input. This study refers also to the modification of the chloride ion penetration measurement technique, which is the novelty of this study. The tests presented in this work were carried out 28 and 180 days after the preparation of the samples, which made it possible to consider the influence of aging on the properties of materials. The interpretation of the obtained results was enriched with the analysis of mercury intrusion porosity (MIP) results, which allowed for drawing reliable conclusions.

## 2. Materials and Methods

The studies were performed on alkali-activated concretes in which FA (Połaniec power plant, Poland) and ground-granulated blast-furnace slag (GGBFS) (Ekocem, Dąbrowa Górnicza, Poland) blends were used as precursors. The oxide compositions of both precursors FA and GGBFS used in this research are provided below (Table 1).

The review of oxide compositions of the precursors published by Osio-Norgaard, Gevaudan, and Srubar [44] shows that, in terms of oxide composition, FA and GGBFS used in this study are similar to the precursors described in the above-mentioned data compilation on ashes and slags. The solution used as an activator for the AA binder preparation was an aqueous solution of sodium silicate Geosil^®^ 34417 with an additional amount of water added. The chemical composition of this solution is presented in Table 2.

The blends of precursors were prepared by the substitution of FA with GGBFS in the following proportions, 5%, 20%, and 35%, expressed as a percentage of FA mass. Referring to the precursor compositions, three concretes were prepared denominated as AAC5, AAC20, and AAC35, respectively.

Design assumptions of the mixtures were based on previous experience of the research team with works on alkali-activated mortars [8,9] and examples available in the literature [42]. The trial batches with blended FA/GGBFS binders (0–40%) were prepared. For each trial material, the workability of the mixture, compressive strength, and chloride ion penetration after 28 days were tested. The optimal FA/GGBFS ratios for final research were chosen based on the obtained results. The trial batches of concretes enabled the establishment of the optimal mixing time, which was 5 min for already-prepared ingredients, as well as the alkali-activated paste content. This experimental approach enabled the adjustment of the amount of paste in concrete at a level of 300 dm^3^/m^3^.

For each of the designed recipes (presented in Table 3), the following characteristics were kept constant: water/binder (w/b) ratio = 0.37; alkaline solution/binder ratio = 0.53; and the amount of paste in concrete = 300 dm^3^/m^3^. Water is the total mass of water added (water added and water contained in Geosil^®^ 34417); binder is a sum of dry components of the precursor FA and GGBFS (by mass); and alkaline solution is the mass of diluted with water Geosil^®^ 34417.

**Figure 1:**
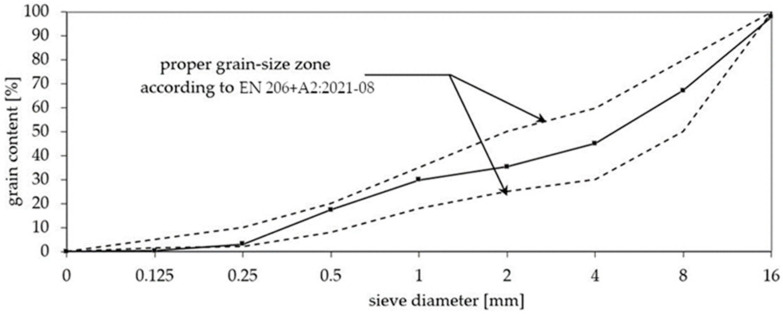
Particle size distribution of AACs.

The coarse aggregates were basalt grains composed to form within the range of the grading curve for the concretes (Figure 1).

Basalt aggregate is a crushed igneous rock with an apparent particle density of 3.17 g/cm^3^ according to EN 1097-6:2013-11 [45], meeting the requirements of the PN-EN 12620+A1:2010 [46] standard. Quartz sand conforming to EN 206+A2:2021-08 [47] was used. The samples were kept in molds under a plastic cover for one day. After that, they were removed and stored in laboratory conditions (18 ± 2 °C; HR = 75%), covered by plastic foil to avoid water evaporation.

The mechanical properties of the hardened AACs were tested on 3 specimens of each material at 28 and 180 days to monitor their evolution over time. Compressive and tensile splitting strength tests were performed according to current standards PN-EN 12390-3 [48], PN-EN 12390-6: 2011 [49], and EN 206+A2:2021-08 [47]. The tests were carried out on cubic samples whose dimensions of the sides were 100 mm.

The most reliable test to determine the resistance to penetration of chlorides is the bulk diffusion test (ASTM C1556-11a [33]). The direct result of this time-consuming measurement is the apparent chloride diffusion coefficient. However, other standards exist that allow a more rapid evaluation of the impact of chloride aggression on concrete. Relations between coefficients obtained by different test methods have been analyzed and presented by Wang and Liu [50].

**Figure 2:**
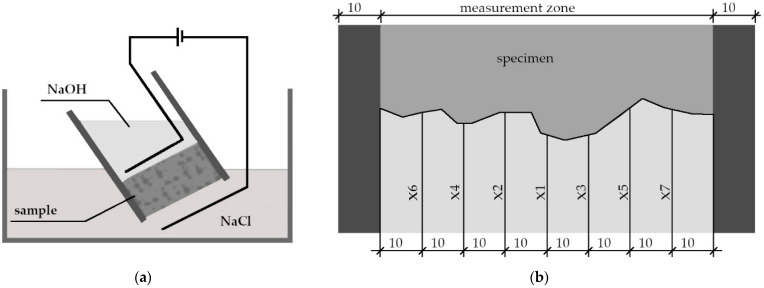
(**a**) NT BUILD 492 migration test scheme; (**b**) principle of chloride penetration depth measurement.

To test the chloride ion penetration of alkali-activated materials, the NT BUILD 492 method [40,51,52] was selected in this study. The significant advantage of this method is the relatively short duration of the measurement. Due to the application of an electric potential, the ion movement across the saturated sample is accelerated. The specimens were initially vacuum saturated with distilled water. In accordance with the recommendations for AAC tests [42], the test procedure was modified using a reduced electric voltage (10 V instead of 60 V). The NT BUILD 492 scheme is presented in Figure 2a. The average chloride penetration depth was determined by silver nitrate solution, as presented in Figure 2b.

Measurements of the chloride ion diffusion coefficient were carried out on samples of 11 cm in diameter and 5 cm in height after 28 and 180 days of maturation to investigate the coefficient changes over time. For each of the materials, chloride penetration depths were measured on 4 splits of the samples. The results obtained were compared with available data [10] to confirm the consistency of the results obtained with other materials with the same type of blended precursors.

It is well known that porosity, pore size distribution, and tortuosity strongly affect mechanical properties and chloride transport in hardened concretes [31,53,54]. Each material was also subjected to a porosity analysis using mercury porosimetry using Poromaster Micromeritics AutoPore IV. For each type of concrete, a sample of size approx. 1 cm^3^ was cut from the cylinder sample, and its total porosity and pore size distribution were determined. Depending on the pressure and amount of mercury injected, the pore diameters and their contents in AACs were specified.

## 3. Results and Discussion

**Figure 3:**
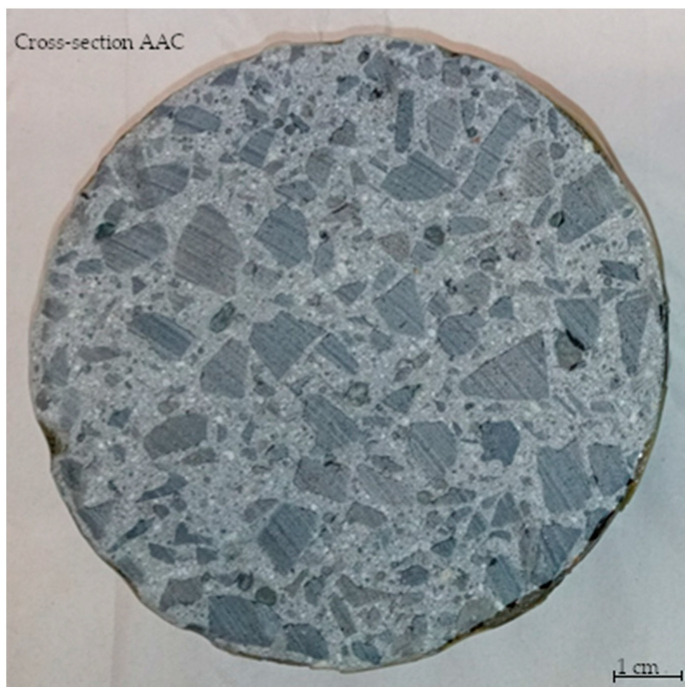
Cross-section of the concrete cylinder sample of 11 cm diameter and 5 cm height.

The apparent density at ambient conditions (temp. 20 ± 2 °C) was measured for 20 specimens of each material. The apparent densities of the tested materials after 180 days were: 2.25 g/cm^3^, 2.30 g/cm^3^, and 2.38 g/cm^3^ for AAC5, AAC20, and AAC35, respectively. The standard deviation was less than 0.05 for each AAC. An example of the test specimen is presented in Figure 3.

### 3.1. Mechanical Characteristics

**Figure 4:**
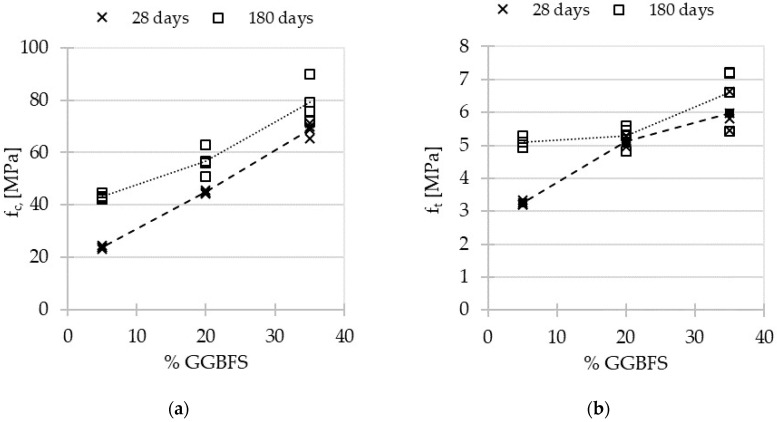
Mechanical properties of alkali-activated concretes after 28 and 180 days: (**a**) compressive strength (f_c_); (**b**) splitting tensile strength (f_t_).

The results of compressive and splitting tensile strength tests of three concretes are presented in Figure 4a,b.

The results of the compressive and tensile strength tests show that the substitution of FA by GGBFS positively influenced the mechanical properties of AACs. The test results indicate an almost linear relationship between GGBFS content and compressive and splitting tensile strength at 28 days, and in particular, a near double increase between AAC5 (23.7 MPa) and AAC20 (44.8 MPa) and an almost triple increase between AAC5 and AAC35 (68.8 MPa). The GGBFS impacts the microstructure by reducing the porosity (see Section 3.3) and therefore improves the mechanical performance.

The obtained values are generally comparable to results observed in the literature for the AACs with similar compositions [43]. Even for materials with the lowest GGBFS content (5%), the obtained value of compressive strength (23.7 MPa) presents the same order of magnitude as fly-ash-based AACs (100% FA) cured at 70 °C (16 MPa) [43]. It was noted that the mechanical strength of the studied concretes can be slightly higher than for mortars with the same amounts of FA and GGBFS [30,55].

Values obtained at 180 days are also in correlation with the increase in GGBFS content. The impact of aging is positive for all the tested concretes, but especially for AAC5 with the lowest GGBFS content: 23.7 MPa at 28 days and 43.2 MPa at 180 days for compressive strength, and 3.25 MPa at 28 days and 5.11 MPa at 180 days for splitting tensile strength. The reason for the reported phenomena is the low reactivity of alkali-activated fly ash [29] and the high reactivity of GGBFS containing calcium, which is responsible for quick early strength development [26].

### 3.2. Chloride Ion Penetration and Diffusion Coefficient

**Figure 5:**
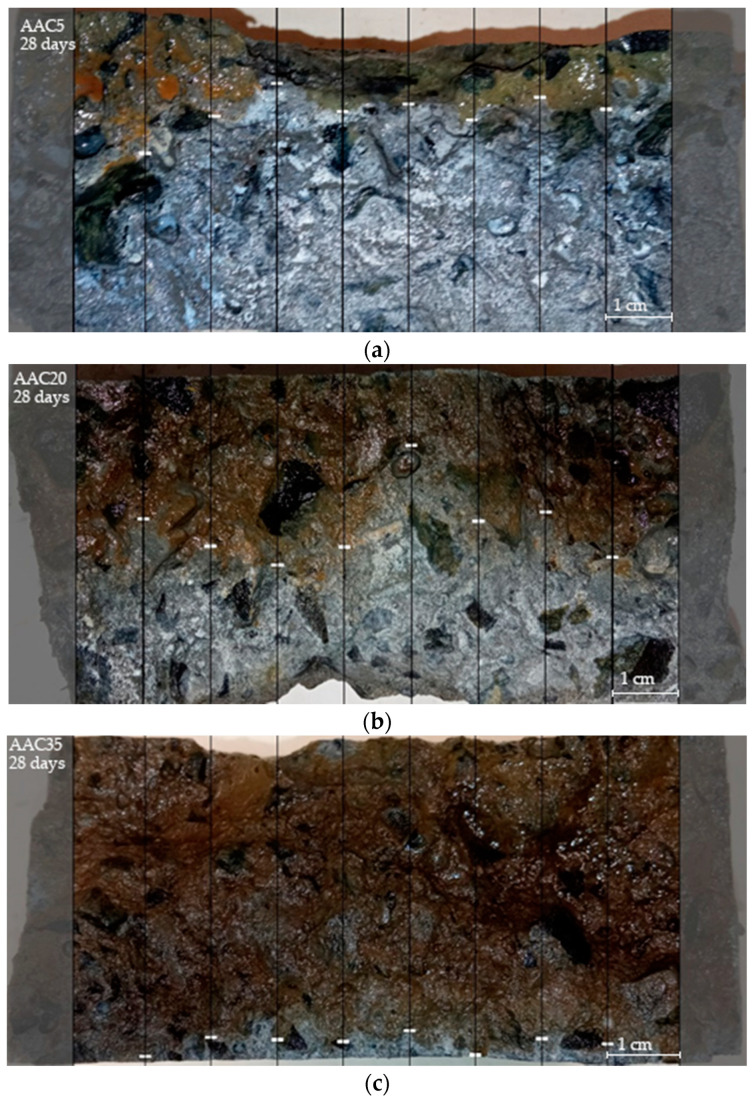
Chloride penetration depths of alkali-activated concretes after 28 days: (**a**) AAC5; (**b**) AAC20; (**c**) AAC35.

**Figure 6:**
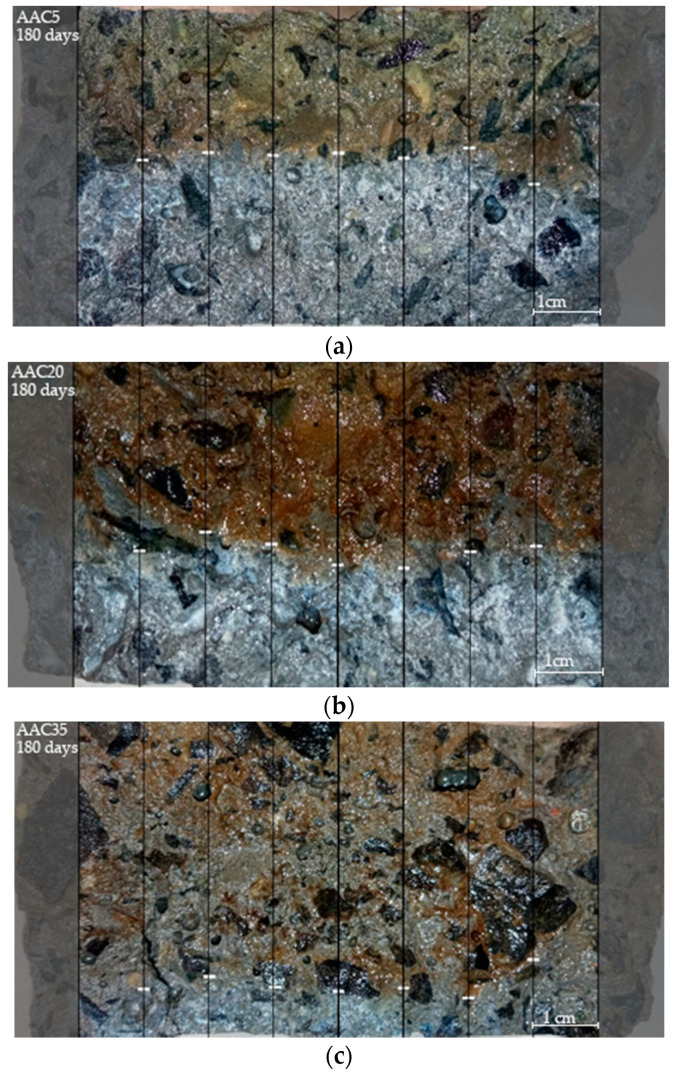
Chloride penetration depths of alkali-activated concretes after 180 days: (**a**) AAC5; (**b**) AAC20; (**c**) AAC35.

After the chloride ion penetration tests were performed, the samples were split and sprayed with silver nitrate AgNO_3_ solution. The examples of the cross-sections of the samples showing the penetration profile are presented in Figure 5 and Figure 6.

The average values and standard deviations of chloride penetration depth for each sample are presented in Table 4. The results of chloride transport in AACs indicate a strong correlation with GGBFS content: the chloride penetration depth for AAC5 is almost twice that of AAC20, which is more than quadruple that of AAC35. Slag’s impact is therefore clearly pointed out.

**Figure 7:**
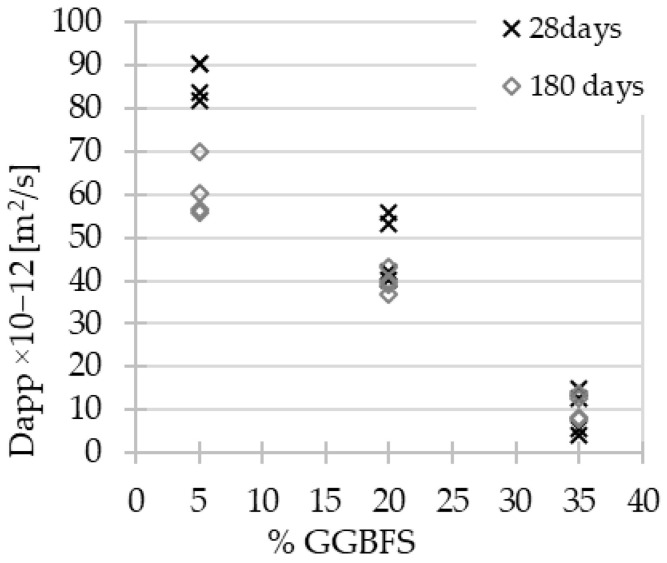
AACs’ chloride diffusion coefficient values vs. GGBFS content.

**Figure 8:**
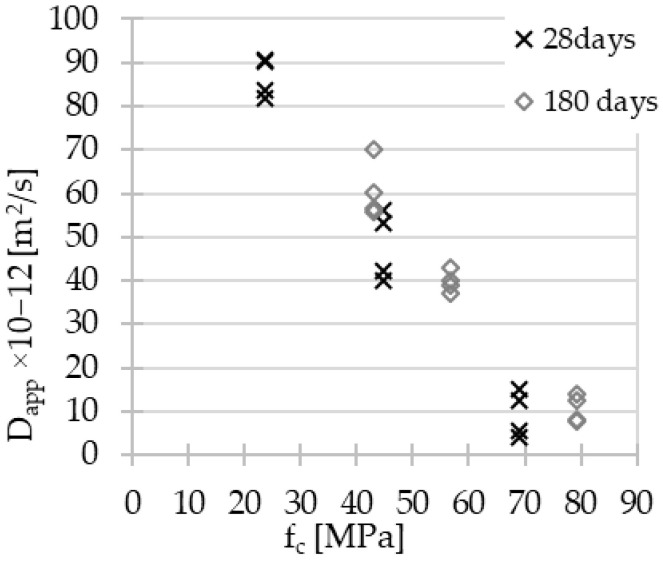
AACs’ chloride diffusion coefficient values vs. f_c_.

The values of the non-steady-state apparent chloride migration coefficients were calculated with the formula 1 [56,57] and are depicted in Figure 7 and Figure 8. The thickness of each specimen was about 50 mm; nevertheless, it was measured precisely for each cylinder. The solution temperature was 20 °C, and the value of applied voltage was 10 V, as already mentioned in the previous section.

(1) $$D_{nssm}=\frac{R\cdot T}{Z\cdot F}\cdot \frac{e}{\Delta E}\cdot \frac{x_{d}-\alpha \sqrt{x_{d}}}{\Delta t}$$

where:

(2) $$\alpha =2\cdot \sqrt{\frac{R\cdot T}{Z\cdot F}\cdot \frac{e}{\Delta E}}\cdot erf^{-1}\left(1-2\frac{c_{d}}{c_{0}}\right)$$

*D_nssm_*: non-steady state migration coefficient, m^2^/s;
*Z*: absolute value of ion valence, for chloride, z = 1;
*F*: Faraday constant, F = 9.648 × 104 J/(V‧mol);
Δ*E*: value of applied voltage, V;
*R*: gas constant, R = 8.314 J/(K‧mol);
*T*: average value of the initial and final temperatures in the anolyte solution, K;
*e*: thickness of the specimen, m;
*x_d_*: average value of the penetration depths, m;
Δ*t*: test duration, seconds;
*erf*^−1^: inverse of error function;
*c_d_*: chloride concretion at which the colour changes, *c_d_* ≈ 0.07 for OPC concrete;
*c*_0_: chloride concentration in the catholyte solution, N.

In the case of the chloride ion penetration coefficient, a strong linear relationship with the material composition was observed, independently of the material’s aging (Figure 7). In parallel, a linear relationship was highlighted between the chloride coefficient and compressive strength (Figure 8). Both relationships should be due to the effect of the porosity decreasing within slag addition [58], which is discussed in the next section. The modification of the test method enables the comparison of results obtained for geopolymer concrete with reference methods dedicated to Portland cement concrete [33,41,42]. The values of chloride diffusion coefficient after 28 days of curing in ambient conditions show the positive influence of GGBFS content increase: 86.6 × 10^−12^ m^2^/s (σ = 3.8), 47.8 × 10^−12^ m^2^/s (σ = 6.8), and 9.4 × 10^−12^ m^2^/s (σ = 4.6) for AAC5, AAC20, and AAC35, respectively. The results obtained for AAC20 and AAC35 were partially comparable to values available in the literature for AAC with close precursors’ compositions [43] and tested by NT Build 492 without modifications. However, the applied modifications [42] made it possible to carry out the test on the AACs with low slag content with satisfying repeatability in contrast to the inconsistency of results available in the literature [34,35,36,55].

Noushini A. [42] defined the superior limit value of the chloride diffusion coefficient for AACs applied in chloride environments equal to 14 × 10^−12^ m^2^/s. This recommendation was based on experimental results according to the standard ASTM C1556 [33]. Values of diffusion coefficient for AAC35, almost regardless of the time seem to be promising even with such strong constraints. However, the rapid test methodology adjusted to AACs requires verification. Indeed, the results obtained are essential to determine the impact of the precursor composition and the material’s age on the resistance to chloride aggression. The transfer of chloride ions in AACs takes place via the pore network of materials. As mentioned before, the low reactivity of FA causes long-term development of AAC’s properties, even for resistance to chloride aggression.

### 3.3. MIP Porosity

**Figure 9:**
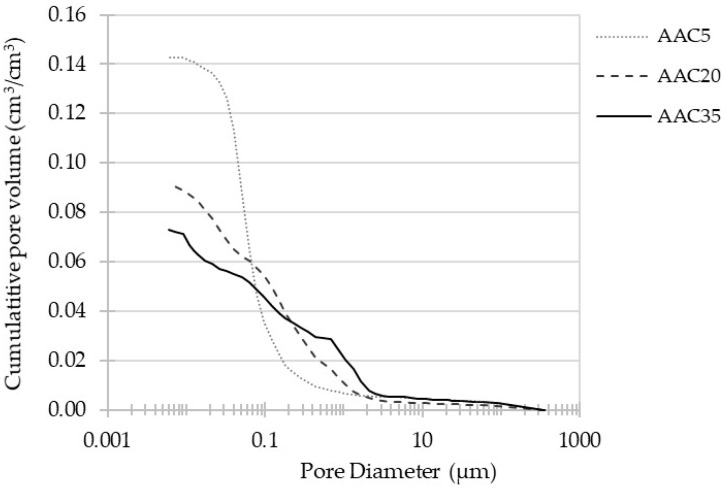
Total porosity of alkali-activated concretes.

**Figure 10:**
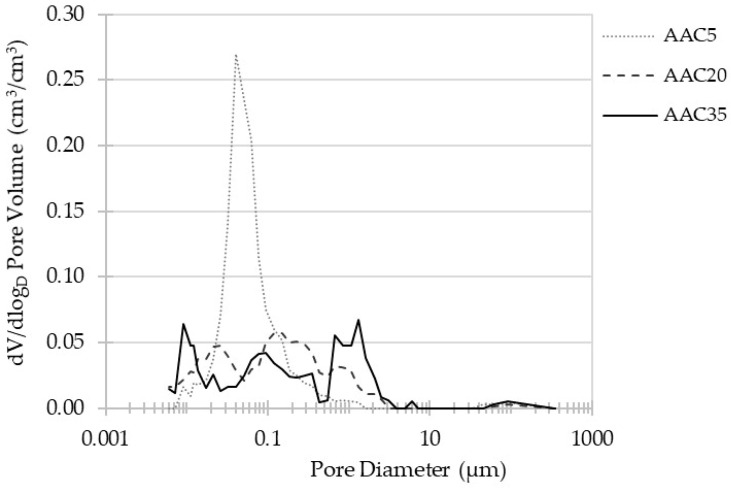
Pore size distribution of alkali-activated concretes.

In the case of porous materials such as cement concrete and AAC, their characteristics are strongly dependent on their open porosity. In order to investigate the total volume of pores and their size distribution, Mercury Intrusion Porosimetry tests were undertaken. The results for three AACs are presented in Figure 9 and Figure 10.

MIP tests were conducted after 180 days of the curing process in ambient conditions (20 ± 2 °C). In terms of total pore content of materials, increasing the level of substitution of FA by GGBFS reduces porosity by almost half: AAC5, AAC20, and AAC35 total porosities are 14.28%, 9.18%, and 7.30%, respectively. A strong association between the results and the precursors’ compositions were reported. The reason for this phenomenon is a difference in hydration products formed through the activation of FA and GGBFS [30]. Due to the high calcium content of GGBFS, the main reaction product of the alkali activation process is C-A-S-H gel, which results in a denser matrix [59]. An increase in slag content in the precursor’s composition results in a reduced ratio of geopolymeric gel (N-A-S-H), generated by the alkali activation of FA, to C-A-S-H gel, which leads to a more homogenous and compact structure [28].

The pore size distribution presented in Figure 10 shows the diversity of the internal structure of the concretes. A distinct difference was observed between AAC5 and other materials. The porosity of AAC5 is dominated by 0.04 µm diameter pores, while AAC20 and AAC35 present uniform and continuous pore diameter distributions. Slight peaks of pores of 0.15 µm diameter for AAC20 were noticed as well as 0.09 µm and 1.32 µm for AAC35. However, AAC20 and AAC35 seem to have similar inner structures. The same tendencies were observed for the microstructure of corresponding materials (mortars) and are discussed in the available literature [60].

Certainly, the variable content of GGBFS in precursor compositions triggered changes in the structure’s development process. Except for the slag addition, the AACs’ components were identical, as well as the hardening and storage conditions. Therefore, the results presented in this paper are closely related to the precursor used. Increasing slag content affected the inner structure of material, but changes are much more significant for AAC5 than AAC20. However, other analysed characteristics were affected with a linear trend.

The results presented in this paper show a strong influence of blended precursor composition on the properties of AAC without temperature curing. Taking into consideration ingredients used for the preparation of concretes, it can be assumed that all of the phenomena were caused by a change in FA/GGBFS ratio, resulting finally in concrete porosity change. Geopolymer gel N-A-S-H, formed from the activation of FA, was gradually replaced by C-A-S-H (from alkali activation of GGBFS), which densified the inner structure of materials by affecting the mechanical strength and chloride aggression resistance. The obtained results show almost linear correlations between mechanical strength and chloride ion penetration of AAC; however, it should be noted that there is still a lack of testing procedures dedicated to alkali-activated concrete. The modification of chloride ion penetration measurement [42] applied for the conducted tests shows the necessity of constant development and is an area of the authors’ further research.

## 4. Conclusions

The conducted research indicates a significant influence of GGBFS addition on the mechanical properties and resistance to chloride aggression of AAC, taking into account the impact of aging of materials. The results of the tests carried out show that it is possible to obtain satisfactory properties for AAC without the need for temperature curing the material. The conducted research allows for drawing general conclusions:

- The addition of GGBFS significantly reduces the porosity of alkali-activated concretes and changes the pore size distribution from one dominated by a specific pore diameter to continuous pore size distribution.
- Increasing quasi-linear relationships between the GGBFS content in the material composition and the compressive strength, splitting tensile strength and the value of the chloride diffusion coefficient have been demonstrated.
- The effect of aging on the improvement of material properties is particularly noticeable in the case of concretes with low GGBFS content.

The presented results indicate the necessity for further research aimed at a better understanding of the mechanism of chloride penetration in AAC and the influence of blast furnace slag addition on the physical and mechanical properties of alkali-activated concretes in view of their durability.

## Figures and Tables

**Table 1:** Chemical compositions of FA and GGBFS used in the presented research.

wt.%	SiO_2_	Al_2_O_3_	FexO_y_	CaO	MgO	SO_3_	K_2_O	Na_2_O	P_2_O_5_	TiO_2_	Mn_3_O_4_	Cl^−^
FA	52.30	28.05	6.32	3.05	1.71	0.28	2.51	0.76	0.69	1.35	0.07	-
GGBFS	39.31	7.61	1.49	43.90	4.15	0.51	0.36	0.47	-	-	-	0.04

**Table 2:** Chemical composition of Geosil^®^ 34417.

Characteristic	Unit	Woellner Geosil^®^ 34417
Na_2_O content	wt.%	16.74
SiO_2_ content	wt.%	27.5
Density	g/cm^3^	1.552
Viscosity	mPa×s	470
Weight ratio (WR = wt.% SiO_2_/wt. Na_2_O)	-	1.64
Molar ratio (MR = mol SiO_2_/mol Na_2_O)	-	1.70

**Table 3:** Composition of alkali-activated concretes [kg/m^3^].

	AAC5B	AAC20B	AAC35B
FA	336.9	292.3	244.1
GGBFS	17.7	73.1	131.4
Alkaline Solution + water	189.4	195.1	200.5
Sand 0/2	662.4	662.4	662.4
Basalt 2/8	708.9	708.9	708.9
Basalt 8/16	648.4	648.4	648.4
water/binder (w/b) ratio	0.37		
alkaline solution/binder ratio	0.53		
amount of paste in concrete	300 dm^3^/m^3^		

**Table 4:** The average values and standard deviations of chloride penetration depth of AAC.

		AAC5	AAC20	AAC35
28 days	Penetration depth [mm]	37.2	19.1	4.1
	σ—standard deviation	1.8	2.3	1.5
180 days	Penetration depth [mm]	28.8	18.3	7.2
	σ—standard deviation	2.3	1.3	1.3
